# MicroRNA-144-3p Inhibits Tumorigenesis of Oral Squamous Cell Carcinoma by downregulating ERO1L

**DOI:** 10.7150/jca.33267

**Published:** 2020-01-01

**Authors:** Xian Li, Yong Li, Chao Jiang, Liang Chen, Ning Gan

**Affiliations:** 1 Stomatological Hospital of Chongqing Medical University, Chongqing, China.; 2 Chongqing Key Laboratory of Oral Diseases and Biomedical Sciences, Chongqing, China.; 3 Chongqing Municipal Key Laboratory of Oral Biomedical Engineering of Higher Education, Chongqing, China.

**Keywords:** miR-144-3p, ERO1L, STAT3, tumorigenesis, OSCC

## Abstract

An increasing number of studies indicate that miR-144-3p is dysregulated in numerous cancers, but its role in oral squamous cell carcinoma (OSCC) remains largely unknown. Herein we demonstrated that miR-144-3p expression was significantly downregulated in OSCC tissues and cell lines. Moreover, the low level of miR-144-3p expression was associated with the clinical characteristics of OSCC patients. Furthermore, ectopic expression of miR-144-3p inhibited the proliferation, migration, and invasion of OSCC cells *in vitro*, and blunted the tumorigenic ability of OSCC cells *in vivo*. Additionally, the levels of miR-144-3p were negatively correlated with the expression status of endoplasmic reticulum oxidoreduction-1-like (ERO1L) in OSCC cell lines. Subsequently, we identified that ERO1L was a direct target of miR-144-3p. Intriguingly, we found that miR-144-3p downregulation of ERO1L inhibited the activity of signal transducer and activator of transcription 3 (STAT3) in OSCC cells. Therefore, miR-144-3p suppresses tumorigenesis by targeting ERO1L/STAT3 signaling pathway in OSCC. miR-144-3p may a candidate target for OSCC treatment.

## Introduction

Oral squamous cell carcinoma (OSCC), accounting for the 90% of oral cancers, is one of the most leading causes of cancer-related mortality worldwide 1. The incidence rate of OSCC is rising, especially in the younger people 2. The etiology of OSCC is complicated and many factors are involved, mainly including alcohol abuse, smoking and virus infection 3. Despite advances in the treatment of OSCC, the prognosis for patients with advanced OSCC remains rather poor 4. Therefore, it is urgent need to identify the molecular biomarkers that can be used as therapeutic targets of OSCC. Previously, we have demonstrated that osteopontin is a promising target for OSCC with hyperactivated mechanistic target of rapamycin (mTOR) 5. Recent years, accumulating evidence unveiled that microRNAs (miRNAs) play a crucial role in the development of many malignancies, including oral cancer 6. However, the mechanisms of miRNAs in the progression of OSCC remain to be fully elucidated.

miRNAs are naturally occurring small non-coding RNAs that function as negative regulators of protein-coding genes in multiple cellular processes such as cell proliferation, differentiation and invasion 7. Through base paring with the 3' untranslated region (3'-UTR) of target messenger mRNAs (mRNAs), miRNAs inhibit their post-transcriptional translation or enhance their cleavage 8. Depending on the genes they regulate, miRNAs can function as either oncogenes or tumor suppressors in tumorigenesis 8. The dysregulation of miR-144-3p has been reported in a wide range of tumor types, including breast cancer, glioblastoma, hepatocellular carcinoma, and et al 9-11. However, the biological role and clinical significance of miR-144-3p in OSCC are still not known.

Endoplasmic reticulum oxidoreduction-1-like (ERO1L) is an oxidoreductase, which plays a critical role in disulfide bond formation of secreted and cell surface molecules 12. Disulfide bonds are required for proper conformation and function of these molecules 13. ERO1L is elevated in many cancers such as gastric cancer, breast cancer, and lung cancer 14-16. It has been shown that ERO1L participates in tumor progression through promotion of cell proliferation, migration, and invasion 14, 16. However, the significance of ERO1L in OSCC is unclear and the regulation of ERO1L expression also remains to be elucidated.

In this study, we found that miR-144-3p expression was significantly downregulated in OSCC tissues and cell lines. Furthermore, its level affected clinicopathological characteristics of OSCC patients. Moreover, miR-144-3p was able to inhibit OSCC cell proliferation, migration and invasion *in vitro* as well as tumor growth *in vivo.* Additionally, we found that ERO1L is a direct target of miR-144-3p and miR-144-3p mediated downregulation of ERO1L resulted in a decrease in the activity of signal transducer and activator of transcription 3 (STAT3) in OSCC cells. Therefore, our study provides the first evidence of the regulatory mechanisms of miR-144-3p on ERO1L/STAT3 signaling pathway in the carcinogenesis of OSCC, which may shed light on their targeted applications in OSCC therapies.

## Materials and Methods

### Clinical samples

Clinical samples of 48 tumor tissues and the adjacent normal tissues of OSCC patients who underwent surgery from December 2015 to June 2018 were obtained from patients undergoing surgical resection in the Affiliated Hospital of Stomatology, Chongqing Medical University. All specimens were diagnosed for primary squamous cell carcinoma of the tongue by more than two pathologists. All the patients provided informed consent to donate their tissues in a prospective study before undergoing surgery. All the patients enrolled were adults (older than 18 years old), with no history of cancer, chemotherapy or radiotherapy, and with access to their clinical data. The clinical data are shown in Table 1. This research was approved by the Ethics Committees of Chongqing Medical University (#2015-10).

### Cell cultures

The normal human oral epithelial cells (HOEC) and the human OSCC cell lines KB, HSC-2, HSC-3, HSC-5, CAL-27, SCC-9, and SCC-15 were obtained from the American Type Culture Collection (ATCC) or the Institute of Biochemistry and Cell Biology of the Chinese Academy of Sciences (Shanghai, China). All cell lines were cultured in DMEM with 10% FBS and 1% penicillin/streptomycin in 5% CO_2_ at 37 °C.

### Lentivirus-Mediated miR-144-3p overexpression

Lentivirus with miR-144-3p overexpressing vector (pGLVH1/GFP+Puro) and the empty vector were obtained from GenePharma (Shanghai, China). KB and CAL-27 cells were cultured in 6-well plates. When the cells reached a confluence of 50-60%, they were infected with the miR-144-3p overexpressing lentiviruses or control lentiviruses at a multiplicity of infection (MOI) of 20 in the presence of 6 μg/ml polybrene (GenePharma). Stable cell lines were selected by using 3 μg/ml puromycin (Sigma) for 2 weeks.

### Quantitative real-time PCR (qRT-PCR)

For the qRT-PCR analysis of miRNAs, total RNA was extracted from tissues and cells using Trizol (Invitrogen) following the producer's guideline. The expression of miR-144-3p was detected using the Hairpin-it miRNAs qPCR Quantitation Kit (GenePharma) according to the manufacturer's protocol. qRT-PCR was performed on the ABI Prism 7500 fast Sequence Detection System (Applied Biosystem). U6 served as an internal control. The experiment was conducted in triplicate and repeated three times. The primers used were as follows: miR-144-3p specific stem-loop reverse transcription primers: 5′-GTCGTATCCAGTGCAGGGTCCGAGGTATTCGCACTGGATACGACAGTACA-3′; miR-144-3p forward, 5′-GGGAGATCAGAAGGTGATT-3′; reverse, 5′-GTGCAGGGTCCGAGGT-3′. U6 forward, 5′-CTCGCTTCGGCAGCACA-3′; reverse, 5′-AACGCTTCACGAATTTGCGT-3′.

### Western blot

Western blotting analyses were performed as described previously 17. In brief, cells were lysed and protein was harvested using RIPA buffer (Beyotime, Haimen, China). Equal amount of the extracts was subjected to SDS-PAGE, transferred onto PVDF membranes, and then protein was detected using primary antibodies specific to ERO1L (Abcam), phosph-STAT3 Tyr^705^ (Cell Signaling), STAT3 (Cell Signaling), phosph-AKT Ser^473^ (Cell Signaling), AKT1 (Cell Signaling) or β-actin (Santa Cruz), and HRP-conjugated secondary antibodies with ECL Western Blotting Substrate (Thermo Scientific).

### Transient transfection

The miR-144-3p mimics (5'-UACAGUAUAGAUGAUGUACU-3'), mimics negative control (5'-UUCUCCGAACGUGUCACGUTT-3'), miR-144-3p inhibitor (5'-AGUACAUCAUCUAUACUGUA-3'), and inhibitor negative control (5'-CAGUACUUUUGUGUAGUACAA-3') were purchased from GenePharma (Shanghai, China). The ERO1L overexpression plasmid was constructed by introducing the human ERO1L cDNA into the pcDNA3.1 vector (Invitrogen), and was designated as pcDNA3.1-ERO1L. Specific siRNA against ERO1L (5'-GCACTGCTCTGAAGATCTT-3') and a scramble siRNA (5'-TTCTCCGAACGTGTCACGT-3') were synthesized by RiboBio (Guangzhou, China). Cells were seeded in 12-well plates and transfected with the above oligonucleotides or vectors using Lipofectamine 3000 (Invitrogen) following the manufacturer's instructions. Cells were harvested at 72 h after transfection and used in subsequent analyses.

### Reporter constructs and luciferase reporter assay

A fragment of ERO1L 3'-untranslated region (3'-UTR) containing the putative binding site for miR-144-3p was amplified from normal human genome DNA and cloned into the Spe I and Hind III sites of pMIR-REPORT firefly luciferase vector (Promega), named as ERO1L-WT. The primer sequences were as follows: forward 5'-GGACTAGTCTCCCTAATATCCTTCAGTGA-3'; reverse 5'-CCAAGCTTTTGAATACAATTTCAGCT-3'. ERO1L mutant 3'-UTR recombinant plasmid was generated using the Quick Change site-directed mutagenesis kit (Promega) with the primers: 5'-GTATGTCACTTAAATCGATGACAATTGTTTTATTTTTC-3' and 5'-GAAAAATAAAACAATTGTCATCGATTTAAGTGACATAC-3', which generated a mutation of 6 bps from TACTGT to ATGACA in the predicted miR-144-3p target binding site, named as ERO1L-Mut. All constructs were confirmed by DNA sequencing.

For reporter assay, cells were incubated in triplicate in 24-well plates and transfected with 200 ng promoter constructs (ERO1L-WT or ERO1L-Mut) together with 50 ng pRL-TK plasmids and 50 nM miR-144-3p mimics or miR-NC. 48 h later, luciferase activity was examined with the Dual-Luciferase Reporter Assay System (Promega). Firefly luciferase activity was normalized to renilla luciferase activity.

### Cell proliferation assay

The cell proliferation was evaluated with MTT assay as described previously 18. In brief, cells were seeded into 96-well plates at a density of 2×10^3^ per well and cultured with DMEM containing 10% FBS. After 1-5 days, the cells were stained with 20 μl of MTT (5 mg/ml in PBS) (Sigma) for 4 h at 37 °C, followed by removal of the culture medium and incubation with 150 μl of dimethyl sulfoxide (DMSO). The absorbance was measured at 570 nm using a microtiter plate reader (Varioskan Flash, Thermo).

### Colony formation assay

Cells were seeded into 10 cm dish at a density of 300 cells per dish and cultured about 3 weeks in DMEM containing 10% FBS. Cell culture was terminated when obvious colonies could be observed in dishes by the naked eye. After removing the media, the cells were washed with PBS three times, fixed with methanol for 15 min, and dyed with 0.1% crystal violet (Sigma) for 20 min. The number of colonies containing over 50 cells was counted using a microscope (Leica Microsystems). All experiments were tested in triplicate, and the mean values of colony number of 3 parallel dishes were calculated.

### Wound healing assay

Cells were seeded into 6‐well plates and then cultured for 24 h until 90% confluence. A scratch was created using a sterile micropipette tip. After removed the cell debris by rinsing twice with serum‐free medium, the cells continued to be incubated in an incubator with complete medium. The scratch healing areas of cells were observed during different time points. Finally, three randomly selected fields were photographed by an inverted microscope (BD Biosciences).

### Transwell assay

A 24‐pore transwell chamber (Corning) with polycarbonate membrane filter covered with the gelatin package was used to measure the invasive ability of cells. After diluted with 100 μl serum‐free medium, Matrigel (BD Biosciences) was used to cover the bottom membranes of transwell chamber. After that, 2 × 10^4^ cells were inoculated onto the upper chamber. The serum and growth factors such as chemokines were added in the lower chamber and incubated overnight. The invasive cells were fixed with 4% paraformaldehyde and then stained with Giemsa stain (Sigma). The invasive cells were counted through a microscope (×200).

### Induction of subcutaneous tumors in nude mice

Subcutaneous tumors were established in nude mice (BALB/c, 5 week old) as described previously 5. In brief, 4×10^6^ CAL-27 cells expressing miR-144-3p or the empty vector in 200 μl of DMEM were subcutaneously inoculated into the right posterior back region and tumor growth was monitored. Tumor volume was measured using a caliper every 4 days. The tumor volume was calculated using the following equation: tumor volume = 1/2 × (length × width^2^). After 33 days, the mice were sacrificed and photographed, and the tumors were harvested and weighed. Six male mice were used in each cohort. All animals were maintained and used in accordance with the guidelines of the Animal Center of Chongqing Medical University.

### Statistical analysis

All statistical analyses were performed using SPSS 19.0 and Graphpad Prism 5.0. The data were analyzed using a 2-tailed paired Student's t-test or a Chi-square test as appropriate. It is statistically significant when *P* < 0.05.

## Results

### miR-144-3p is downregulated in OSCC tissues and cell lines

**Figure 1:**
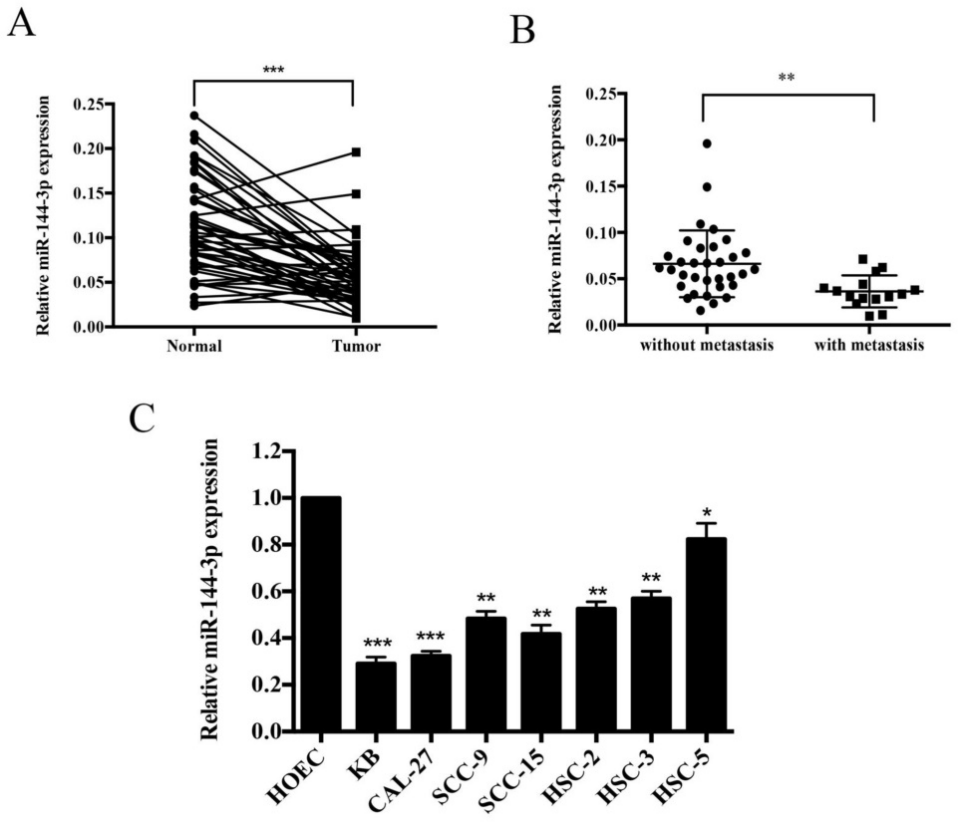
** miR-144-3p is significantly downregulated in OSCC tissues and cancer cell lines. (A)** qRT-PCR analysis of miR-144-3p in OSCC cancer tissues and adjacent normal tissues. **(B)** Relative expression levels of miR-144-3p in OSCC cancer tissues with (n=15) and without lymph node metastasis (n=33). **(C)** Relative expression levels of miR-144-3p in OSCC cell lines (KB, HSC-2, HSC-3, CAL-27, SCC-9, HSC-5, and SCC-15) and a normal human oral epithelial cell line, HOEC. *P<0.05; **P<0.01; ***P<0.001.

qRT-PCR was performed to investigate the expression abundance of miR-144-3p in OSCC tissues and cell lines. As shown in Figure 1A, miR-144-3p expression was remarkably lower in the tumor tissues than that of adjacent normal tissues (n=48). Moreover, as depicted in Figure 1B, miR-144-3p was apparently lower in the patients with lymph node metastasis (n=15) than in those without lymph node metastasis (n=33). Furthermore, miR-144-3p levels were significantly decreased in the human OSCC cell lines (KB, HSC-2, HSC-3, HSC-5, CAL-27, SCC-9, and SCC-15) compared with those of the normal human oral epithelial cells, HOEC (Figure 1C). In addition, the clinicopathological significance of miR-144-3p in OSCC patients has been investigated and summarized in Table 1. The results showed that miR-144-3p was significantly associated with larger tumor size, lymph node metastasis, and advanced TNM stage. Taken together, miR-144-3p is remarkably downregulated in OSCC tissues and cell lines, and correlated with the clinical characteristics of OSCC patients.

### The suppressive effect of miR-144-3p on cell proliferation, migration and invasion *in vitro*

**Figure 2:**
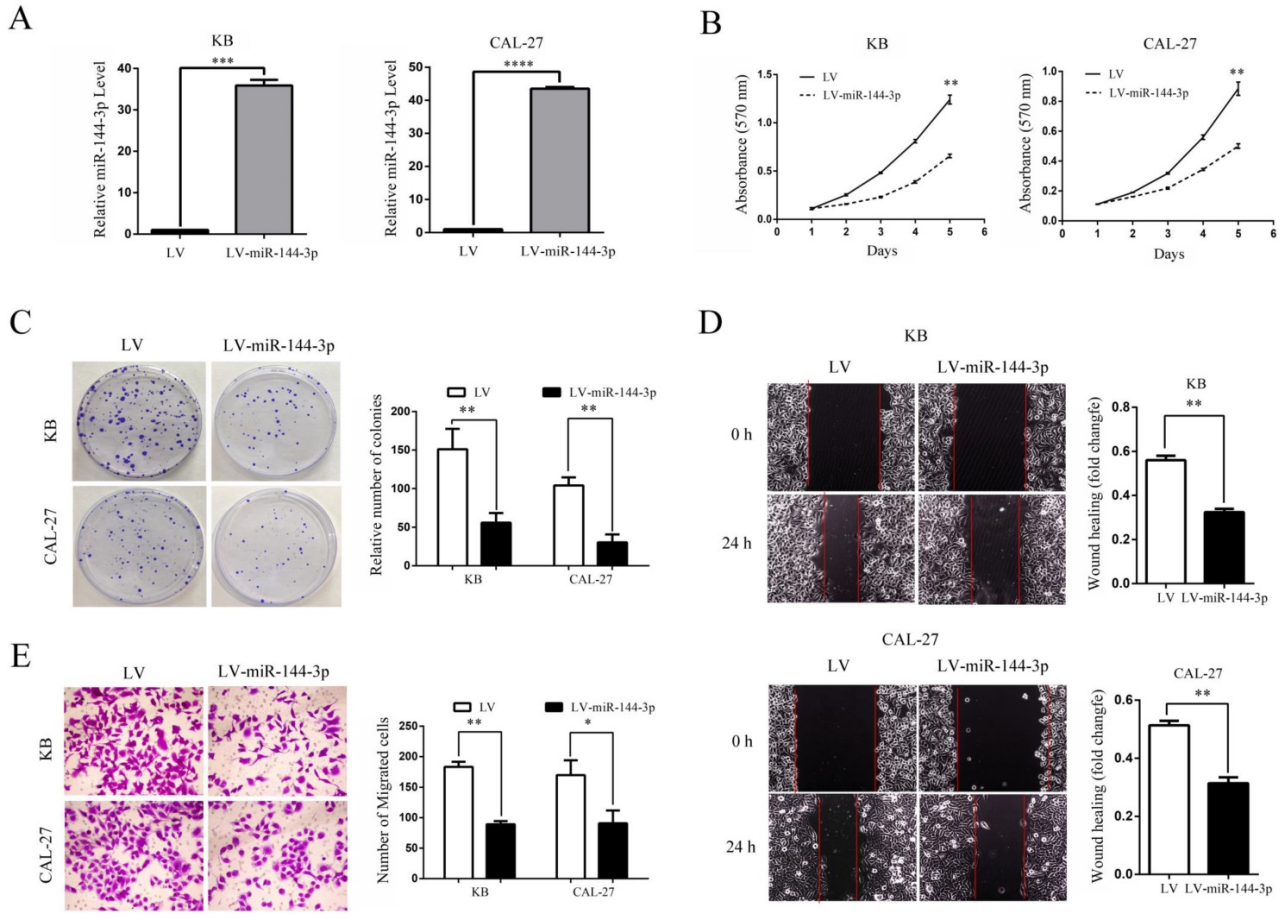
** Ectopic expression of miR-144-3p inhibits OSCC cell growth, migration, and invasion *in vitro*. (A-E)** KB or CAL-27 cells were infected with lentivirus harboring a vector encoding miR-144-3p (LV-miR-144-3p) or the empty vector (LV). **(A)** qRT-PCR analysis of miR-144-3p levels of the indicated cells. **(B)** Cell proliferation of the indicated cells was examined using an MTT assay. **(C)** The colonies formed by the indicated cells were stained and counted. Representative images (left panel) and quantifications (right panel). **(D)** Migration of the indicated cells was examined using the wound healing assay. Representative images (left panels) and quantifications (right panels). **(E)** Invasion of the indicated cells was examined using the transwell assay. Representative images (left panel) and quantifications (right panel). Data are means ± SD for triplicate samples from one of three representative experiments. *P<0.05; **P<0.01; ***P<0.001.

Since miR-144-3p expression was significantly negative associated with tumor size and lymph node metastasis in OSCC, we speculated that miR-144-3p may exert suppressive effects on cell proliferation and invasion of OSCC cells. Thus, KB and CAL-27 cells which showed relatively lower miR-144-3p expression were infected with lentiviruses for overexpressing miR-144-3p. Successful overexpression of miR-144-3p in KB and CAL-27 cells was confirmed by qRT-PCR (Figure 2A). As expected, the ectopic expression of miR-144-3p markedly suppressed the proliferation of KB and CAL-27 cells, as demonstrated by MTT assay (Figure 2B) and colony formation assay (Figure 2C). Moreover, we examined the influence of miR-144-3p on cell migratory and invasive capacities. As shown in the Figure 2D and 2E, the wound healing and transwell assays demonstrated that overexpression of miR-144-3p significantly inhibited cell migration and invasion both in KB and CAL-27 cells. Taken together, these data suggested that miR-144-3p functions as a tumor suppressor in OSCC cells.

### miR-144-3p inhibits tumor growth of OSCC *in vivo*

**Figure 3:**
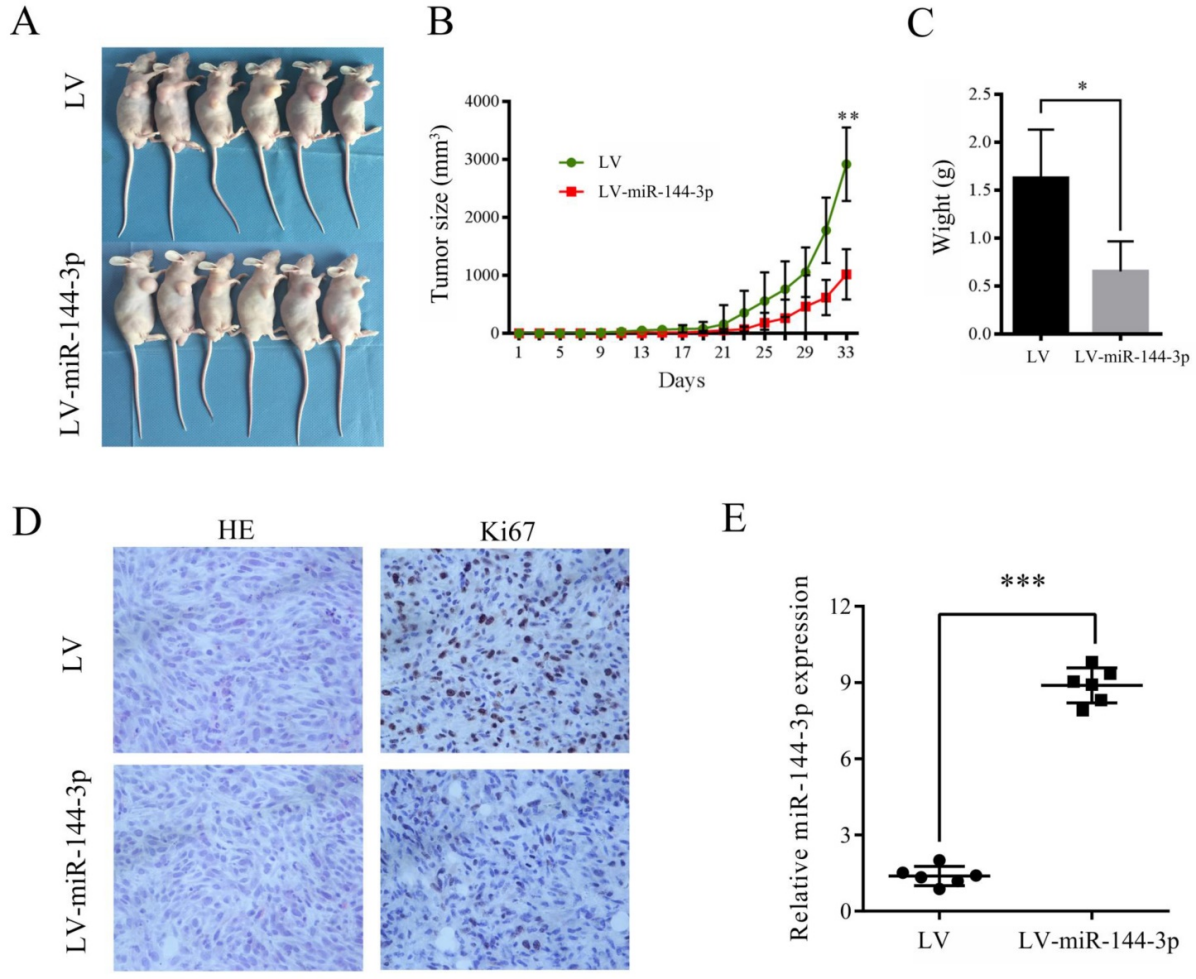
** Ectopic miR-144-3p expression inhibits tumor growth *in vivo*. (A-E)** CAL-27 cells transduced with LV-miR-144-3p or LV lentiviruses were inoculated subcutaneously into nude mice, following by monitoring for tumor growth. **(A)** Tumor pictures. **(B)** Tumor volumes at different times. **(C)** Tumor weight. **(D)** H&E and immunohistochemical staining of the indicated tumor tissues (×200). Representative images were presented. **(E)** qRT-PCR analysis of miR-144-3p levels of the indicated tumor tissues. *P<0.05; **P<0.01; ***P<0.001.

To investigate the role of miR-144-3p in the growth of OSCC cells *in vivo,* CAL-27 cells transfected with lentiviral vector encoding miR-144-3p or empty vector were subcutaneously injected into the right anterior armpit of nude mice, and then the tumor growth was monitored. As depicted in Figure 3A-C, overexpression of miR-144-3p significantly suppressed the tumorigenic capacity of CAL-27 cells. Furthermore, IHC analysis revealed that tumor tissues derived from mice with injection of miR144-3p-overexpressing CAL-27 cells exhibited much weaker staining for Ki-67 than those in the control group (Figure 3D). Moreover, increased miR-144-3p expression in the tumor tissues derived from these nude mice was confirmed by qRT-PCR (Figure 3E). Therefore, these data indicate that miR-144-3p suppresses the tumorigenic ability of OSCC cells *in vivo*.

### ERO1L is a direct target of miR-144-3p

**Figure 4:**
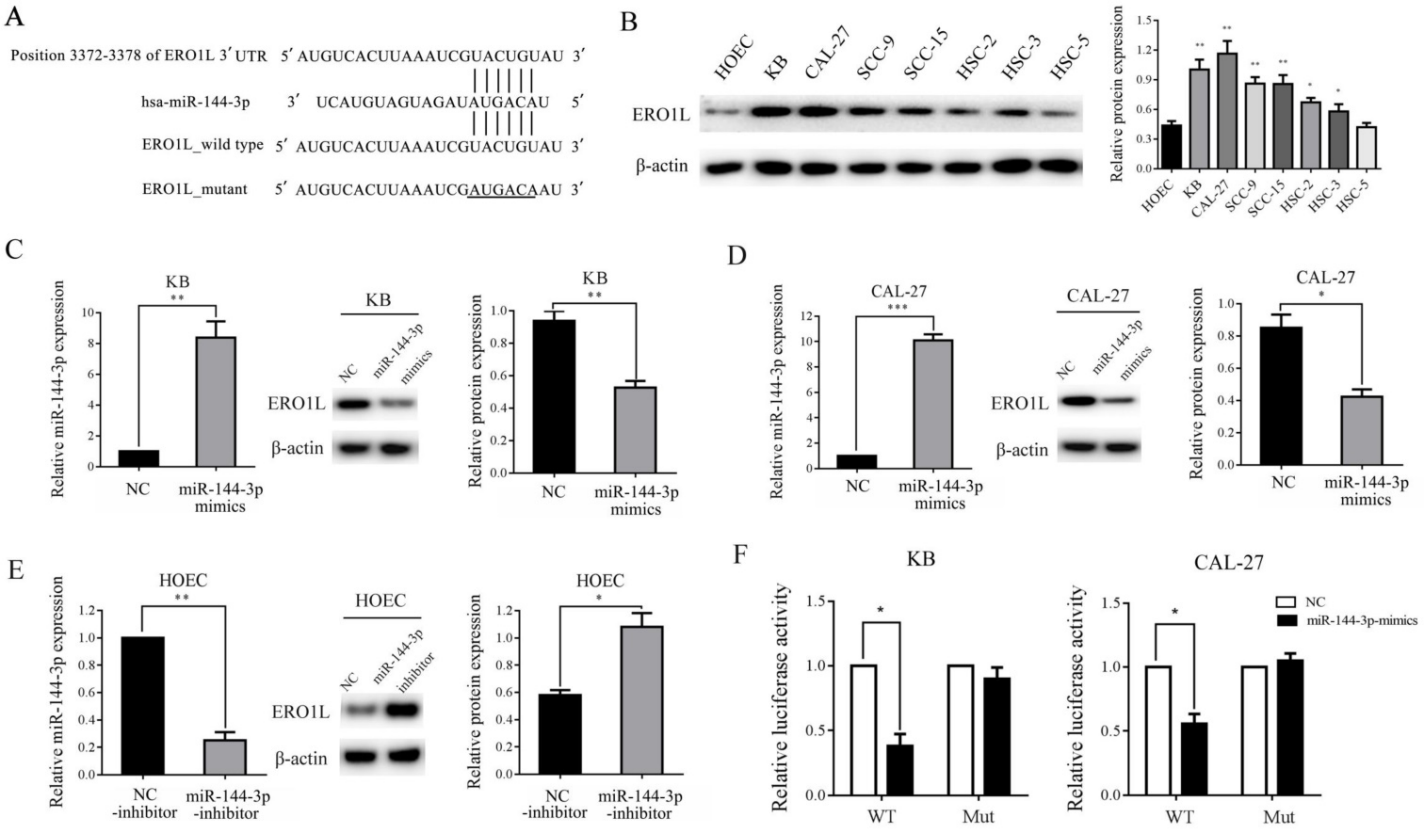
** ERO1L is a direct target of miR-144-3p. (A)** Sequence alignment of predicted miR-144-3p within the ERO1L 3'UTR and its mutated sequence for luciferase reporter assay. **(B)** The expression of ERO1L in the indicated cells was examined by western blotting (left panel) and densitometric analysis of this experiment normalized to β-actin is shown (right panel). **(C and D)** KB and CAL-27 cells were transfected with the miR-144-3p mimics or negative control (NC). **(E)** HOEC cells were transfected with the miR-144-3p inhibitor or negative control (NC-inhibitor). **(C-E)** The level of miR-144-3p of the indicated cells was analyzed by qRT-PCR (left panels). The expression of ERO1L in the indicated cells was examined by western blotting (middle panels). Quantitation of relative band intensity was normalized to β-actin by scanning densitometry (right panels). **(F)** Luciferase reporter assay was performed in KB (left panel) and CAL-27 (right panel) cells that were co-transfected with miR-144-3p mimics or NC together with reporter vectors containing ERO1L 3' UTR or mutated ERO1L 3' UTR. Relative luciferase activities are presented. Data indicate mean ± SD of triplicate samples from one of three representative experiments. *P<0.05; **P<0.01; ***P<0.001.

To investigate the underlying molecular mechanisms of miR-144-3p in the growth and metastasis of OSCC cells, we searched for the putative target genes of miR-144-3p using bioinformatics tools, such as TargetScan, miRanda, and PicTar. Because miR-144-3p was able to inhibit the proliferation and invasion of OSCC cells, we focused on the genes that promoted tumor growth and metastasis. The analysis of the 3'-UTR of the ERO1L mRNA revealed a potential binding site for miR-144-3p, which indicated that the existence of a regulative relationship between miR-144-3p and ERO1L (Figure 4A). Furthermore, western blot analysis demonstrated that the expression of ERO1L is significantly upregulated in OSCC cell lines as compare with the control cells, and the protein level of ERO1L is negatively correlated with miR-144-3p levels in OSCC cells (Figure 4B). These data prompted us to examine whether or not miR-144-3p can inhibit the expression of the endogenous ERO1L protein in OSCC cells. As depicted in the left panel of Figure 4C, transfection of miR-144-3p mimics into KB cells resulted in a substantial increase of miR-144-3p expression compared to miR-NC-transfected cells. As expected, overexpression of the miR-144-3p markedly reduced the endogenous ERO1L protein levels in KB cells (Figure 4C middle panel and right panel). A similar result was obtained in CAL-27 cells when miR-144-3p were overexpressed (Figure 4D). In contrast, transfection of miR-144-3p inhibitor led to a significant decrease in miR-144-3p expression as well as markedly upregulated expression of ERO1L in HOEC cells (Figure 4E). Therefore, ERO1L is a downstream effector of miR-144-3p.

To further assess whether ERO1L is a direct target of miR-144-3p, a luciferase activity assay was performed. A 639 base pair fragment of the 3'UTR (ERO1L-WT) and the mutant 3'UTR (ERO1L-Mut) were cloned into the pMIR-REPORT vector. The KB and CAL-27 cells were co-transfected with ERO1L-WT or ERO1L-Mut, miR-144-3p mimics or NC, and pRL-TK luciferase reporters. As shown in Figure 4F, miR-144-3p was able to markedly decrease the relative luciferase activity of ERO1L-WT in both the KB and CAL-27 cells, whereas that in the cells transfected with ERO1L-Mut was not reduced. Collectively, above data suggested that ERO1L is a direct downstream target of miR-144-3p.

### miR-144-3p modulates OSCC progression through inhibition of ERO1L/STAT3 signaling pathway

**Figure 5:**
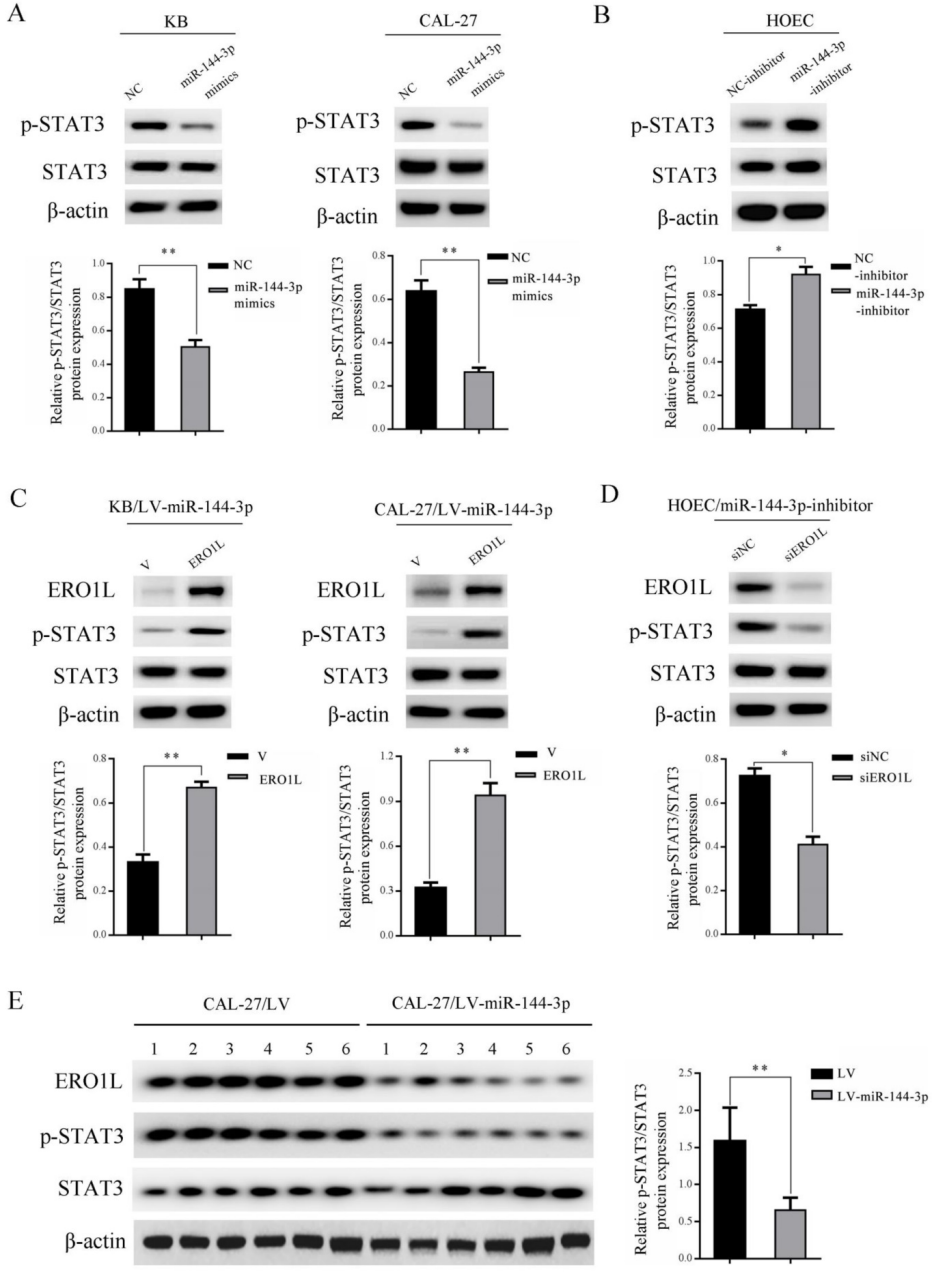
** miR-144-3p mediated downregulation of ERO1L led to suppression of STAT3 activity. (A)** KB (left panels) and CAL-27 (right panels) cells were transfected with the miR-144-3p mimics or negative control (NC). **(B)** HOEC cells were transfected with the miR-144-3p inhibitor or negative control (NC-inhibitor). **(C)** LV-miR-144-3p lentiviruses infected KB (left panels) and CAL-27 (right panels) cells were transfected with pcDNA3.1-ERO1L or the empty vector pcDNA3.1 (V) for 48 h. **(D)** miR-144-3p inhibitor transduced HOEC cells were transfected with the control siRNAs (siNC) or siRNAs targeting ERO1L for 48 h. **(A-D)** Cell lysates were subjected to immunoblotting with the indicated antibodies (upper panels). The relative quantitation of p-STAT3 determined by scanning densitometry analysis upon normalization for STAT3 (lower panels). **(E)** Tumor tissues derived from CAL-27 cells transduced with LV-miR-144-3p or LV lentiviruses were subjected to immunoblotting with the indicated antibodies (left panel) and densitometric analysis of this experiment normalized to STAT3 is shown (right panel). Data indicate mean ± SD of triplicate samples from one of three representative experiments. *P<0.05; **P<0.01.

STAT3 has been shown to play a critical role in the development of OSCC 19. To further investigate whether the STAT3 signaling pathway is involved in the regulation of OSCC mediated by miR-144-3p, western blot experiments were performed. As shown in Figure 5A, ectopically expressed miR-144-3p mimics led to a markedly downregulated expression of phosphorylated STAT3 Tyr^705^ (p-STAT3) in both KB (left panels) and CAL-27 cells (right panels). Conversely, inhibition of miR-144-3p in HOEC cells by transfection of miR-144-3p inhibitor led to a significant increase in p-STAT3 (Figure 5B). To further establish the functional regulation of ERO1L/STAT3 signaling axis by miR-144-3p, we ectopically expressed ERO1L in miR-144-3p-overexpressing KB cells. As shown in the left panels of Figure 5C, overexpression of ERO1L reactivated the compromised STAT3 activity mediated by miR-144-3p expression in KB cells. Consistent results were obtained in miR-144-3p-overexpressing CAL-27 cells when ERO1L was overexpressed (Figure 5C right panels). Furthermore, we used ERO1L siRNAs to deplete ERO1L in miR-144-3p suppressed HOEC cells. Western blot analysis demonstrated that depletion of ERO1L attenuated the elevated STAT3 activity driven by miR-144-3p inhibition (Figure 5D). Moreover, decreased expression of p-STAT3 and a concomitant downregulation of ERO1L were observed in tumor tissues derived from miR-144-3p-overexpressing CAL-27 cells compared to the corresponding control cells (Figure 5E). Collectively, these data revealed that miR-144-3p downregulation of ERO1L led to inhibition of STAT3 activity in OSCC cells.

## Discussion

Accumulating evidences showed that dysregulation of miRNAs was frequently observed in various types of cancers and plays an important role in tumor progression 8. The biological roles of miRNAs in OSCC remain incompletely characterized. Herein, we found that miR-144-3p was dramatically downregulated in OSCC tissues and cells, and its low expression was significantly associated with the clinical characteristics of OSCC patients. Further studies demonstrated that miR-144-3p inhibited OSCC cell proliferation, migration and invasion *in vitro* as well as tumor growth *in vivo*. Moreover, ERO1L was identified to be a direct and functional target of miR-144-3p. In addition, we showed that miR-144-3p downregulation of ERO1L led to inhibition of STAT3 in OSCC cells.

Recent years, the expression status and biological function of miR-144-3p have been intensively investigated in many cancers 9, 20-22. However, previous studies have produced inconsistent results. For example, Yin reported that miR-144-3p is downregulated in breast cancer and functions as a tumor suppressor through repressing CEP55 9. Liu et al. showed that the expression of miR-144-3p is decreased in lung adenocarcinoma (LUAD) tissues, and overexpression of miR-144-3p inhibited propagation and invasiveness of LUAD cells 20. In contrast, other groups reported that miR-144-3p is upregulated in the specimens of nasopharyngeal carcinoma and clear cell renal cell carcinoma and serves as an onco-miRNA 21, 22. In the current study, we found that miR-144-3p is downregulated in 77% of the OSCC tissue samples compared with the adjacent normal tissues. Interestingly, inconsistent with our findings, a previous study performed using an Indian cohort comprising 61 OSCC tumors and 9 independent normal oral tissues showed that miR-144-3p is upregulated in OSCC 23. This discrepancy can be attributed to several effects such as racial and etiological differences. We further showed that miR-144-3p is significantly decreased in OSCC cell lines compared with the normal oral epithelial cells. Subsequent function assays showed that overexpression of miR-144-3p inhibited the proliferation, migration, and invasion of OSCC cells *in vitro* and suppressed the tumor growth of OSCC cells *in vivo*. Therefore, our data indicated that miR-144-3p exerts a tumor suppressive function in the development of OSCC. In addition, we found that the low expression of miR-144-3p was associated with larger tumor size, lymph node metastasis, and advanced tumor stage of OSCC, suggesting that it may be used as a diagnostic marker in the pathological processes of OSCC.

Recently, a couple of genes have been identified to be regulated by miR-144-3p in tumors 10, 11, 24, 25. For example, Wu et al. reported that SGK3 is a direct target of miR-144-3p in hepatocellular carcinoma 11; Lan and colleagues have reported that c-Met is a downstream target of miR-144-3p in glioblastoma 10. In the present study, to investigate the underlying mechanisms of miR-144-3p in OSCC, we screened for the target genes of miR-144-3p and found that ERO1L is a direct and functional target of miR-144-3p. ERO1L is upregulated in many cancers 14-16, 26. It has been shown that ERO1L can promote tumor progression through acceleration of cell proliferation and invasion 13. However, there is limited documentation on the regulation of ERO1L expression. Only several transcription factors, such as HIF1ɑ and CHOP have been indicated to be involved in the transcriptional regulation of ERO1L 27, 28, while its posttranscriptional regulation remains largely unclear. In the current study, we showed that overexpression of miR-144-3p mimics led to downregulation of ERO1L expression, while inhibition of endogenous miR-144-3p resulted in upregulation of ERO1L. Mechanistic investigations further revealed that that miR-144-3p inhibits the expression of ERO1L through binding the 3'UTR of ERO1L mRNA. To the best of our knowledge, this is the first study to report the posttranscriptional regulation of ERO1L in OSCC. Moreover, our results indicate that ERO1L is involved in the progression of OSCC and appears to be a novel therapeutic target for OSCC. Future works are required to elucidate the clinical significance of ERO1L in OSCC.

Numerus studies indicated that STAT3 is implicated in tumor cell proliferation, invasion, and metastasis in human cancers 29, 30. Upon activation by upstream signals, STAT3 undergoes phosphorylation (Tyr^705^), homo-dimerization, nuclear translocation, and then promotes the transcription of various downstream target genes by directly interacts with their STAT3 binding sites 31. The STAT3 signaling has been reported to be aberrantly activated in OSCC 19. However, the upstream activators of STAT3 remain not fully elucidated. Here we demonstrated that ectopic expression of ERO1L increased p-STAT3, while knockdown of ERO1L led to downregulation of p-STAT3 expression, which suggest that there may be a putative link between activation of STAT3 signaling and ERO1L overexpression in OSCC cells. A previous study showed that depletion of ERO1L led to dramatically attenuation of AKT activity in gastric cancer cells 14. Given that STAT3 is suggested to be a downstream effector of AKT 32, 33 and miR-144-3p negatively regulated the activity of AKT (Supplemental Figure 1), it is thus likely that miR-144-3p downregulation of ERO1L inhibits the activation of STAT3 through suppression of AKT activity. Future studies are needed to explore this possibility.

In conclusion, miR-144-3p serves as a tumor suppressor in OSCC cells by directly targeting the ERO1L/STAT3 pathway. This study not only provides new insight into the mechanism of OSCC, but also suggests that miR-144-3p may be a candidate target for treatment of OSCC.

## Supplementary Material

Supplementary figure S1.

## Figures and Tables

**Table 1:** Correlations between miR-144-3p expression and the clinical characteristics in OSCC patients

Characteristic	Case	miR-144-3p expression		X²	P-value
		Low	High		
**Age (years)**					
≤50	23	11	12	0.083	0.773
>50	25	13	12		
**Gender**					
Male	30	17	13	1.422	0.233
Female	18	7	11		
**Tumour size**					
≤4 cm	28	10	18	5.486	0.019
>4 cm	20	14	6		
**Differentiation**					
High/Moderate	17	9	8	0.091	0.763
Poor	31	15	16		
**Clinical stage**					
Ⅰ-Ⅱ	26	9	17	5.371	0.020
Ⅲ-Ⅳ	22	15	7		
**Lymph node metastasis**					
No	33	12	21	7.855	0.005
Yes	15	12	3		
**Location of the tumors**					
Buccal cancer	21	9	12	0.762	0.383
Tongue cancer	27	15	12		
**Tobacco use**					
No	23	12	11	0.083	0.773
Yes	25	12	13		
**Alcohol use**					
No	16	7	9	0.375	0.540
Yes	32	17	15		

## References

[1] Bray F, Ferlay J, Soerjomataram I, Siegel RL, Torre LA, Jemal A (2018). Global cancer statistics 2018: GLOBOCAN estimates of incidence and mortality worldwide for 36 cancers in 185 countries. CA Cancer J Clin.

[2] Gupta N, Gupta R, Acharya AK, Patthi B, Goud V, Reddy S (2016). Changing Trends in oral cancer - a global scenario. Nepal J Epidemiol.

[3] Kumar M, Nanavati R, Modi TG, Dobariya C (2016). Oral cancer: Etiology and risk factors: A review. J Cancer Res Ther.

[4] Wang B, Zhang S, Yue K, Wang XD (2013). The recurrence and survival of oral squamous cell carcinoma: a report of 275 cases. Chin J Cancer.

[5] Gan N, Zou S, Hang W, Yang D, Zhang X, Yin Y (2017). Osteopontin is Critical for Hyperactive mTOR-Induced Tumorigenesis in Oral Squamous Cell Carcinoma. J Cancer.

[6] Santosh AB, Jones T, Harvey J (2016). A review on oral cancer biomarkers: Understanding the past and learning from the present. J Cancer Res Ther.

[7] Rupaimoole R, Slack FJ (2017). MicroRNA therapeutics: towards a new era for the management of cancer and other diseases. Nat Rev Drug Discov.

[8] Hayes J, Peruzzi PP, Lawler S (2014). MicroRNAs in cancer: biomarkers, functions and therapy. Trends Mol Med.

[9] Yin Y, Cai J, Meng F, Sui C, Jiang Y (2018). MiR-144 suppresses proliferation, invasion, and migration of breast cancer cells through inhibiting CEP55. Cancer Biol Ther.

[10] Lan F, Yu H, Hu M, Xia T, Yue X (2015). miR-144-3p exerts anti-tumor effects in glioblastoma by targeting c-Met. J Neurochem.

[11] Liu T, Liu Y, Miller M, Cao L, Zhao J, Wu J (2017). Autophagy plays a role in FSTL1-induced epithelial mesenchymal transition and airway remodeling in asthma. Am J Physiol Lung Cell Mol Physiol.

[12] Zito E (2015). ERO1: A protein disulfide oxidase and H2O2 producer. Free Radic Biol Med.

[13] Tanaka T, Kutomi G, Kajiwara T, Kukita K, Kochin V, Kanaseki T (2016). Cancer-associated oxidoreductase ERO1-alpha drives the production of VEGF via oxidative protein folding and regulating the mRNA level. Br J Cancer.

[14] Seol SY, Kim C, Lim JY, Yoon SO, Hong SW, Kim JW (2016). Overexpression of Endoplasmic Reticulum Oxidoreductin 1-alpha (ERO1L) Is Associated with Poor Prognosis of Gastric Cancer. Cancer Res Treat.

[15] Tanaka T, Kutomi G, Kajiwara T, Kukita K, Kochin V, Kanaseki T (2017). Cancer-associated oxidoreductase ERO1-alpha promotes immune escape through up-regulation of PD-L1 in human breast cancer. Oncotarget.

[16] Han F, Xu Q, Zhao J, Xiong P, Liu J (2018). ERO1L promotes pancreatic cancer cell progression through activating the Wnt/catenin pathway. J Cell Biochem.

[17] Jin F, Jiang K, Ji S, Wang L, Ni Z, Huang F (2017). Deficient TSC1/TSC2-complex suppression of SOX9-osteopontin-AKT signalling cascade constrains tumour growth in tuberous sclerosis complex. Hum Mol Genet.

[18] Ji S, Lin W, Wang L, Ni Z, Jin F, Zha X (2017). Combined Targeting of mTOR and Akt Using Rapamycin and MK-2206 in The Treatment of Tuberous Sclerosis Complex. J Cancer.

[19] Mali SB (2015). Review of STAT3 (Signal Transducers and Activators of Transcription) in head and neck cancer. Oral Oncol.

[20] Liu C, Yang Z, Deng Z, Zhou Y, Gong Q, Zhao R (2018). Downregulated miR-144-3p contributes to progression of lung adenocarcinoma through elevating the expression of EZH2. Cancer Med.

[21] Xiao W, Lou N, Ruan H, Bao L, Xiong Z, Yuan C (2017). Mir-144-3p Promotes Cell Proliferation, Metastasis, Sunitinib Resistance in Clear Cell Renal Cell Carcinoma by Downregulating ARID1A. Cell Physiol Biochem.

[22] Zhang LY, Ho-Fun Lee V, Wong AM, Kwong DL, Zhu YH, Dong SS (2013). MicroRNA-144 promotes cell proliferation, migration and invasion in nasopharyngeal carcinoma through repression of PTEN. Carcinogenesis.

[23] Manikandan M, Deva Magendhra Rao AK, Arunkumar G, Manickavasagam M, Rajkumar KS, Rajaraman R (2016). Oral squamous cell carcinoma: microRNA expression profiling and integrative analyses for elucidation of tumourigenesis mechanism. Mol Cancer.

[24] Li B, Zhang S, Shen H, Li C (2017). MicroRNA-144-3p suppresses gastric cancer progression by inhibiting epithelial-to-mesenchymal transition through targeting PBX3. Biochem Biophys Res Commun.

[25] Yu M, Lin Y, Zhou Y, Jin H, Hou B, Wu Z (2016). MiR-144 suppresses cell proliferation, migration, and invasion in hepatocellular carcinoma by targeting SMAD4. Onco Targets Ther.

[26] Kutomi G, Tamura Y, Tanaka T, Kajiwara T, Kukita K, Ohmura T (2013). Human endoplasmic reticulum oxidoreductin 1-alpha is a novel predictor for poor prognosis of breast cancer. Cancer Sci.

[27] Rao J, Zhang C, Wang P, Lu L, Qian X, Qin J (2015). C/EBP homologous protein (CHOP) contributes to hepatocyte death via the promotion of ERO1alpha signalling in acute liver failure. Biochem J.

[28] May D, Itin A, Gal O, Kalinski H, Feinstein E, Keshet E (2005). Ero1-L alpha plays a key role in a HIF-1-mediated pathway to improve disulfide bond formation and VEGF secretion under hypoxia: implication for cancer. Oncogene.

[29] Yu H, Lee H, Herrmann A, Buettner R, Jove R (2014). Revisiting STAT3 signalling in cancer: new and unexpected biological functions. Nat Rev Cancer.

[30] Zha X, Wang F, Wang Y, He S, Jing Y, Wu X (2011). Lactate dehydrogenase B is critical for hyperactive mTOR-mediated tumorigenesis. Cancer Res.

[31] O'Shea JJ, Schwartz DM, Villarino AV, Gadina M, McInnes IB, Laurence A (2015). The JAK-STAT pathway: impact on human disease and therapeutic intervention. Annu Rev Med.

[32] Abdelhamed S, Ogura K, Yokoyama S, Saiki I, Hayakawa Y (2016). AKT-STAT3 Pathway as a Downstream Target of EGFR Signaling to Regulate PD-L1 Expression on NSCLC cells. J Cancer.

[33] Kim E, Kim M, Woo DH, Shin Y, Shin J, Chang N (2013). Phosphorylation of EZH2 activates STAT3 signaling via STAT3 methylation and promotes tumorigenicity of glioblastoma stem-like cells. Cancer Cell.

