# Supplementary material for: MicroRNA-144-3p Inhibits Tumorigenesis of Oral Squamous Cell Carcinoma by downregulating ERO1L
Source: J Cancer. 2020 Jan 1;11(3):759–68. doi: 10.7150/jca.33267 (PMC6959053; doi:10.7150/jca.33267)
Supplement: Supplementary file 1 — Supplementary figure S1. [file jcav11p0759s1.pdf]

Figure S1

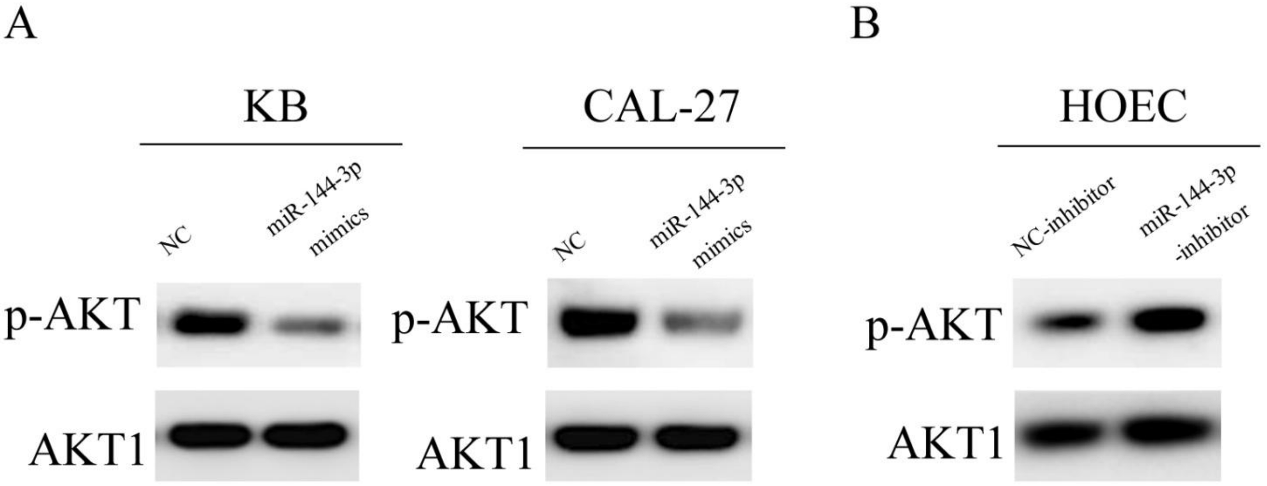

Fig. S1. miR-144-3p inhibits AKT activity.

(A) KB and CAL-27 cells were transfected with the miR-144-3p mimics or negative control (NC). (B) HOEC cells were transfected with the miR-144-3p inhibitor or negative control (NC-inhibitor). (A and B) The expression of p-AKT in the indicated cells was examined by western blotting.
